# Anthocyanin/Honey-Incorporated Alginate Hydrogel as a Bio-Based pH-Responsive/Antibacterial/Antioxidant Wound Dressing

**DOI:** 10.3390/jfb14020072

**Published:** 2023-01-29

**Authors:** Faezeh Lotfinia, Mohammad-Reza Norouzi, Laleh Ghasemi-Mobarakeh, Mohammadreza Naeimirad

**Affiliations:** 1 Department of Textile Engineering, Isfahan University of Technology, Isfahan 84156-83111, Iran; 2 Department of Materials and Textile Engineering, Faculty of Engineering, Razi University, Kermanshah 67144-14971, Iran; 3 Institute für Textiltechnik, RWTH Aachen University, Otto-Blumenthal-Straße 1, 52074 Aachen, Germany

**Keywords:** pH-responsive, antioxidant, honey, anthocyanin, alginate hydrogel, antibacterial wound dressing

## Abstract

Infection is a major problem that increases the normal pH of the wound bed and interferes with wound healing. Natural biomaterials can serve as a suitable environment to acquire a great practical effect on the healing process. In this context, anthocyanin-rich red cabbage (Brassica oleracea var. capitata F. rubra) extract and honey-loaded alginate hydrogel was fabricated using calcium chloride as a crosslinking agent. The pH sensitivity of anthocyanins can be used as an indicator to monitor possible infection of the wound, while honey would promote the healing process by its intrinsic properties. The mechanical properties of the hydrogel film samples showed that honey acts as a plasticizer and that increasing the incorporation from 200% to 400% enhances the tensile strength from 3.22 to 6.15 MPa and elongation at break from 0.69% to 4.75%. Moreover, a water absorption and retention study showed that the hydrogel film is able to absorb about 250% water after 50 min and retain 40% of its absorbed water after 12 h. The disk diffusion test showed favorable antibacterial activity of the honey-loaded hydrogel against both Gram-positive and Gram-negative *Staphylococcus aureus* and *Escherichia coli*, respectively. In addition, the incorporation of honey significantly improved the mechanical properties of the hydrogel. 2,2-diphenyl-1-picrylhydrazyl (DPPH) assay proved the antioxidant activity of the honey and anthocyanin-containing hydrogel samples with more than 95% DPPH scavenging efficiency after 3 h. The pH-dependent property of the samples was investigated and recorded by observing the color change at different pH values of 4, 7, and 9 using different buffers. The result revealed a promising color change from red at pH = 4 to blue at pH = 7 and purple at pH = 9. An in vitro cell culture study of the samples using L929 mouse fibroblast cells showed excellent biocompatibility with significant increase in cell proliferation. Overall, this study provides a promising start and an antibacterial/antioxidant hydrogel with great potential to meet wound-dressing requirements.

## 1. Introduction

Skin is a physical/chemical barrier that protects the internal tissues of the human body from pathogens and dehydration [1]. However, as the largest organ, the skin is also the most frequently injured part of the body. Skin wounds usually heal spontaneously through a complex process [2]. This process is divided into four different phases of (i) hemostasis, which starts immediately after injury; (ii) inflammation, which starts shortly after the hemostasis phase; (iii) proliferation, which begins some days after injury and includes the main healing processes; and (iv) remodeling, in which scar tissue is formed and may last up to several months [3]. 

Severe infections in chronic wounds, such as diabetic foot ulcers, can even lead to death if not properly treated [2]. In the initial stage of wound infection, Gram-positive bacteria dominate, whereas in later stages, Gram-negative bacteria are also present [4]. Bacterial contamination of the wound bed leads to a continuous abnormal inflammation response. Moreover, keratinocytes remain hyperproliferative while re-epithelialization is hindered. The extracellular matrix and granulation tissue break down, which is due to inadequate infiltration of fibroblasts as well as a high level of matrix metalloproteases. Additionally, neovascularization does not occur sufficiently, and fibrin cuffs inhibit the vessels to get oxygen through the wound [5]. Statistics from Europe show that eight million patients are affected by diabetic foot ulcers and up to 450,000 amputations are performed each year, costing more than two billion euros [6]. Therefore, in addition to the natural wound-healing process, the use of an appropriate wound dressing could be helpful as an aid to repair [7].

Bio-based materials have gained increasing importance as wound dressings due to their intrinsic properties, such as biodegradability, biocompatibility, and bioactive properties. Antibacterial, cell-proliferative, and immunomodulatory properties of bio-based materials have been confirmed and reported in several studies [8,9]. 

As a natural bio-based material, honey has been used for wound care application due to its intrinsic properties for thousands of years [10]. Honey consists of lipids, carbohydrates, proteins, vitamins, amino acids, and minerals [11]. These nutrients have a great influence on the wound-healing process. The wound-healing properties of honey are due to its high osmolality, acidity, and non-peroxidic factors and phenols [10]. Clinical trials and research have shown that honey is effective in eliminating microbial infection and reducing reactive oxygen species (ROS). Honey could promote the debridement of the wound and accelerate the repair process. Moreover, the presence of honey positively influences the cell responses of fibroblasts, endothelial, and keratinocytes. Consequently, honey accelerates reepithelization and speed up the wound closure [12,13,14]. 

Alginate is a natural biopolymer composed of mannuronic acid and guluronic acid units. The non-toxicity, biocompatibility, biodegradability and non-immunogenicity of alginate make it a perfect candidate for biomedical applications [15]. It has been used to fabricate various types of alginate-based wound dressings to improve the efficiency of wound care [16,17]. In general, alginate meets the requirements of a moist environment, which is an essential factor for an ideal wound dressing, and absorbs wound exudate so that the skin recovers faster and wound healing is promoted [18]. Alginate is particularly advantageous for encapsulating various active ingredients due to its chemical structure, which consists of hydroxyl and carboxyl groups [16]. Besides, the deprotonated carboxyl groups in the glucuronic acid units are capable of being easily crosslinked by divalent cations [19]. Alginate hydrogels are usually prepared with Ca^2+^ as crosslinking agent. Guluronate blocks participate in crosslinking with calcium cations and form the gel network [20]. Alginate hydrogel-type wound dressings are particularly popular for wound treatment due to their high water content, permeability, and elasticity. In addition, calcium alginate hydrogels possess a hemostatic property that is beneficial for the aggregation of erythrocytes and platelets [21]. Barnett et al. studied the wound healing properties of alginate dressing on a partial full thickness wound model for a period of 14 days. Their results proved the effective hemostatic properties of alginate and a provoked cellular reaction without occlusion [22]. 

Red cabbage (Brassica oleracea var. capitata F. rubra) is rich in bioactive constituents such as anthocyanins, glucosinolates, and flavonols, which are beneficial for human health [23]. Anthocyanins are water-soluble pigments in blue, red, purple, and orange colors found in various fruits and vegetables [24]. Considering the health-promoting function and nontoxicity of anthocyanins, more and more attention is paid to these polyphenols. Antioxidant and anti-inflammatory properties of anthocyanins have been identified in various studies as the main reason for these various health-promoting functions [25]. These properties could be beneficial for accelerating the wound-healing process by reducing the ROS level which results in cell migration and angiogenesis [26]. Moreover, it has been reported that the pH of the medium can change the intensity and shade of anthocyanins. For example, experiments have shown that anthocyanins exhibit red or orange shades at acidic pH values, while increasing pH results in a purple color [27]. 

Although the management of wounds depends on etiology and wound bed features, the usual wound management consists of changing the wound dressing frequently and assessing the wound exudate in terms of color and odor [28]. Evidence suggests that frequent dressing changes may damage the newly grown tissue and increase the risk of infection. The development of a non-invasive monitoring method would be a breakthrough in this case, preventing unnecessary dressing changes [29,30]. Different markers consisting of temperature, matrix metalloproteinase (MMP), and pH have been used to evaluate the wound status [31,32]. Wound exudate pH is one of the parameters that can be used as bio-marker to reflect the existing wound status. As a chronic wound goes through different stages of healing, the pH of the wound exudate changes due to the change in composition [33]. The pH increases during the granulation phase and then falls to 4–6 during normal healing. In contrast, during impaired healing, the pH rises to basic values of 7–9 in various cases. This enormous difference between the two types of wound healing can therefore be used for detection [34].

The present study was aimed at preparing a novel bio-based pH-responsive/antibacterial/antioxidant hydrogel using natural elements for potential application as a wound dressing. For this purpose, honey as a natural product with antibacterial and wound-healing properties and red cabbage extract with antioxidant and pH-responsive properties were incorporated into sodium alginate hydrogel. The mechanical, antibacterial and antioxidant properties of the hydrogel were investigated. Finally, the biocompatibility of the fabricated biomaterial was evaluated.

## 2. Materials and Methods

### 2.1. Materials

Iran Kurdistan honey was used in this study. Sodium alginate (SA, low viscosity), 2,2-Diphenyl-1-picrylhydrazyl (DPPH), phosphate buffer saline (PBS), Roswell Park Memorial Institute (RPMI) medium, calcium chloride (CaCl_2_), methanol, acetone, and buffer solutions with pH values of 4, 7, and 9 were supplied from Sigma-Aldrich (Germany). *Staphylococcus aureus* (*S. aureus*) ATCC 25923, *Escherichia coli* (*E. coli*) ATCC 25922, and L929 mouse fibroblast cells were obtained from Pasture Institute (Tehran, Iran).

### 2.2. Extractions of Red Cabbage Anthocyanins

In order to obtain red cabbage extract (RCE), the procedure of Patras et al. was followed [35]. Briefly, fresh red cabbage was rinsed and cut into small pieces. A portion of 100 g was placed in the blender (Vitamix E310, Vita-Mix, Cleveland, OH, USA) and ground for 1 min in the presence of 200 mL of water as solvent and transferred to the extraction flask. The solid/solvent ratio was adjusted to 1/10 by adding 800 mL of water. The extraction was carried out in a water bath at 37 °C for 30 min with shaking. Finally, it was filtered and stored in a dark bottle for further use.

### 2.3. Preparation of Sodium Alginate Hydrogel Films

The aqueous solution of 8% (*w*/*v*) SA was prepared by dissolving SA in distilled water. Thereafter, 400% (*w*/*w*) honey (with respect to SA) was added to the solution and magnetically stirred for 24 h. The CaCl_2_ solution was added dropwise to SA/honey with stirring to reach a final concentration of 36 mM. Subsequently, 3 mL of red cabbage extract was added and stirred for another 15 min. Finally, the hydrogel solution was poured into a Petri dish and dried at room temperature for 24 h to obtain hydrogel films.

### 2.4. Characterization of the Hydrogel Films

#### 2.4.1. Mechanical Properties

The mechanical properties of the hydrogel film samples were investigated using a uniaxial tensile testing machine (Zwick 1446-60, ZwickRoell, Ulm, Germany) according to ISO 527-3 (2018) standard tensile test method at a crosshead speed of 1 mm/min under ambient conditions. All specimens were cut into 10 × 1.5 cm^2^ strips and mounted on the gripping units, leaving a gage length of 10 cm. The film thickness was measured at different points on the films.

#### 2.4.2. Water Uptake 

In order to measure the water uptake ability of the hydrogel films, the samples were cut into 1 × 1 cm^2^ pieces, dried at 60 °C for 24 h, and weighed (W_0_). Each sample was immersed in 10 mL of water at 37 °C. At specified time intervals the swollen sample was withdrawn, and after removing the excess surface water with a filter paper, the weights of the samples were measured (W_s_) and the process was repeated until the swelling equilibrium was reached. Equation (1) was used to define the water content (WC):

WC % = [(W_s_ − W_0_)/W_0_] (1)

#### 2.4.3. Water Retention

The water retention capacity (WR) of the hydrogel films was investigated. In this regard, a hydrogel film sample was cut into 1 × 1 cm^2^ squares and immersed in deionized water until swelling equilibrium was reached. The sample was then removed, the excess surface water was wiped off with a filter paper, and the initial weight (W_0_) was measured. The sample was then stored at 60% relative humidity and room temperature, and the weight (W_t_) was measured over a 24 h period. Equation (2) was established to calculate the WR %:

WR % = (W_t_/W_s_) × 100 (2)

### 2.5. Fourier Transform Infrared Spectroscopy (FTIR)

The FTIR technique (BOMEM; Hartmann & Braun, Canada) was used to investigate the chemical structure of the hydrogel films. FTIR spectra were recorded in the range of 400–4000 cm^−1^ at a wavenumber resolution of 8 cm^−1^.

### 2.6. Antioxidant Activity Assay

The DPPH assay was used to study the antioxidant properties of hydrogel film samples according to the method of Banerjee et al. [36]. Briefly, 5 mg samples of hydrogel films were immersed in 3 mL of the 100 µM DPPH solutions in methanol and stored in the dark for 30 min. The absorbance at 517 nm was recorded using a UV-vis spectrophotometer (UVmini-1240, Shimadzu, Kyoto, Japan). The degradation of DPPH was calculated according to Equation (3), where A_c_ and A_s_ are the recorded absorbance of the positive control and sample at 517 nm, respectively.

DPPH scavenging = [(A_c_ − A_s_)/A_c_] × 100 (3)

For the time-dependent assay, 5 mg of the sample was added to 3 mL of 100 µM DPPH, and the absorbance at 517 nm was read at different time intervals.

### 2.7. Antibacterial Study

The qualitative disk diffusion assay was used to evaluate the antibacterial activity of the hydrogel film samples against Gram-negative (*E. coli*) and Gram-positive (*S. aureus*) bacteria. For this purpose, a bacterial suspension of 1 × 10^5^ CFU/mL was inoculated on the agar plates using the spreading method, and the hydrogel film samples were carefully placed on them. The plates were incubated at 37 °C for 24 h and the formation of a zone of inhibition around each sample was measured using three replicates. 

### 2.8. pH-Sensitive Color Change

Different buffers with pH values of 4, 7, and 9 were used to study the pH-responsive color change of the prepared hydrogel films. Samples were placed in the petri dishes containing the appropriate buffer for 5 min and photographs were taken.

### 2.9. Cell Viability Assay

The viability of L929 mouse fibroblast cells on the hydrogel films was assessed using colorimetric MTT method. Cells were maintained in RPMI containing 10% fetal bovine serum (FBS) and 1% penicillin/streptomycin at 37 °C and 5% CO_2_ in an incubator (APN-150 CO_2_, Padideh Nogen, Tehran, Iran). Before cell seeding, samples were cut into 1 cm diameter circles and sterilized under a UV lamp for 2 h. Then, the samples were incubated in a 12-well plate with cells at a density of 10^4^ cells/well for 72 h at 37 °C and 5% CO_2_. Subsequently, the wells were washed with PBS and 100 µL of MTT at a concentration of 5 mg/mL was added to each well. After incubating the wells for 4 h at 37 °C and the formation of purple formazan crystals, the medium was removed and 200 µL of DMSO was added to each well to dissolve the crystals. Finally, the concentration of dissolved color in DMSO was measured using an ELISA plate reader (Sunrise, Bio Tek, Winooski, VT, USA), and the viability of the cells was calculated.

### 2.10. Statistical Analysis

All results were statistically analyzed using a one-way analysis of variance (ANOVA), and the significance level was set at 0.05. Measurements were performed in triplicate, averaged, and expressed as mean ± SD.

## 3. Results and Discussion

### 3.1. Characterization of Hydrogel Film Samples

**Figure 1:**
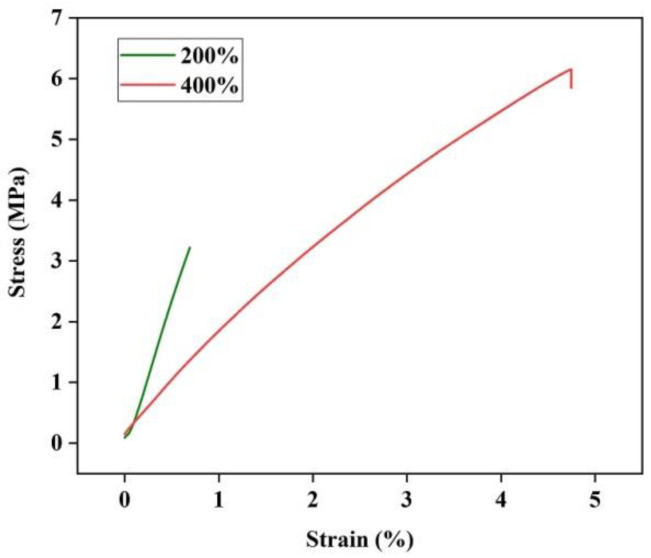
Stress–strain curve and Young’s modulus of the honey-incorporated hydrogel films.

Dehydration of crosslinked alginate films results in strong films that require plasticizers. The addition of plasticizers reduces the intermolecular forces between the polymer chains, which increases the flexibility and mobility of the chains. It is reported that the addition of a plasticizer to crosslinked alginate film improves its handling by reducing brittleness [37]. The stress–strain curve of the honey-incorporated and crosslinked hydrogel film (CA/H) is shown in Figure 1. Moreover, the elastic modulus, tensile strength and elongation at break for the corresponding specimens are listed in Table 1. Increasing the amount of honey from 200% to 400% resulted in a significant decrease (*p* < 0.05) in the elastic modulus, indicating that honey acts as a plasticizer. However, both tensile strength and elongation at break were improved by increasing the amount of honey. It can be concluded that at a honey concentration of 200%, the corresponding hydrogel film is still brittle, resulting in low tensile strength and elongation at break. Moreover, increasing the honey concentration beyond 400% was technically not possible due to the high adhesion of the film. Therefore, the concentration of 400% honey was used during this study.

**Figure 2:**
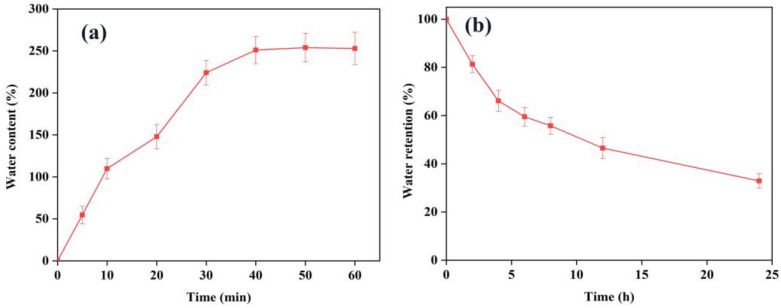
(**a**) Water uptake ability and (**b**) water retention capacity of the hydrogel film.

One of the most important characteristics of an ideal wound dressing is its ability to absorb excess exudate while maintaining a moist wound environment [38]. Accordingly, the evaluation of water content and water retention are important concepts to characterize for wound-dressing materials. The water-uptake ability of honey and RCE-loaded calcium alginate hydrogel film (CA/H/RCE) was investigated. The kinetics of water uptake of CA/H/RCE is shown in Figure 2a. The equilibrium state of water absorption was reached after 50 min, showing the high absorption capacity of the sample when exposed to wound exudate. In addition, the water retention capacity of CA/H/RCE was measured to determine the extent of water loss upon contact with air. Figure 2b demonstrates that the CA/H/RCE sample has adequate water retention capacity and retains more than 40% of its water content after 12 h in air.

### 3.2. FTIR Study

**Figure 3:**
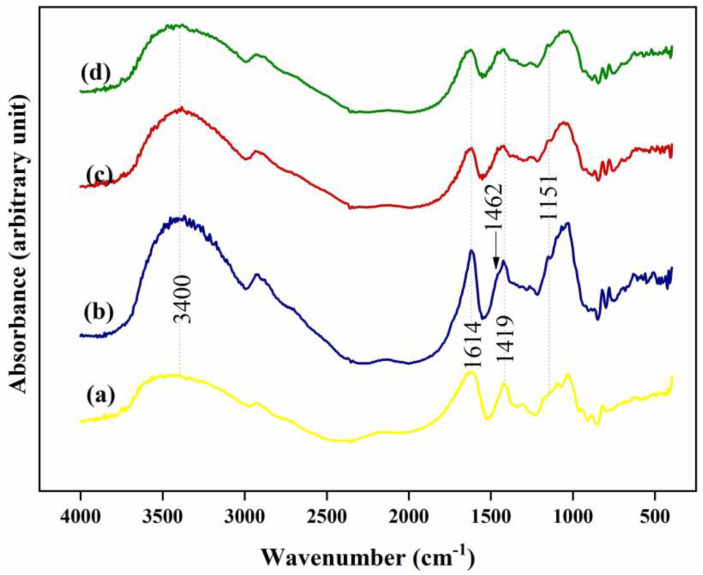
FTIR spectra of: (a) SA, (b) Honey-loaded SA, (c) CA/H, and (d) CA/H/RCE hydrogel film samples.

The interaction between SA, honey, calcium chloride and RCE was investigated by FTIR analysis. Figure 3 shows the resulting FTIR spectra of the different samples. The FTIR spectrum of SA represents the characteristic peaks at 1419 and 1614 cm^−1^, which attribute to the symmetric and asymmetric stretching vibrations of the carboxylate group, respectively. In addition, a broad band of the stretching vibration of the hydroxyl group is observed at 3400 cm^−1^. Considering the spectrum of the honey-loaded SA, the appearance is similar to the spectrum of SA. However, the height ratio of the O-H stretching band at 3400 cm^−1^ to the peak of the carboxylate group at 1614 cm^−1^ is much larger. This can be justified by the presence of the hygroscopic compounds in honey, which carry multiple hydroxyl groups. Moreover, a shoulder at 1462 cm^−1^ was revealed, which is attributed to the stretching vibration of methylene (CH_2_) groups of carbohydrates and/or C=C of aromatic rings in honey. Besides, a new peak at 1151 cm^−1^ is revealed corresponding to C-O stretching in phenolic groups of honey.

Looking at the spectrum of CA/H, the symmetric stretching band of the carboxylate group is shifted to a higher wavenumber, confirming the formation of ionic bonds between divalent calcium ions and carboxylate groups in the alginate [39]. 

**Figure 4:**
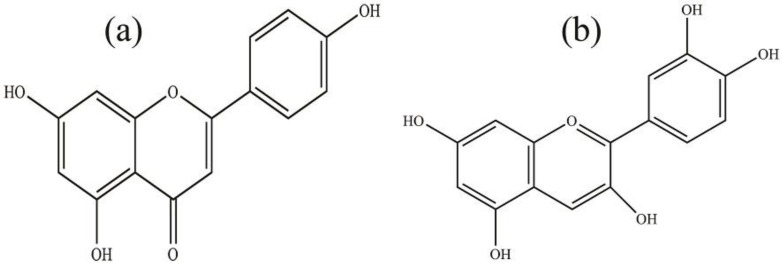
Chemical structure of: (**a**) anthocyanin and (**b**) apigenin.

Taking the spectrum of the CA/H/RCE hydrogel film into consideration, no change can be observed compared to the CA/H spectrum. RCE is a rich source of anthocyanins. Based on the chemical structure of anthocyanin (Figure 4a), it is very similar to one of the flavonoids of honey, apigenin (Figure 4b) [40,41]. Moreover, regarding the high loading percentage of honey (400% with respect to SA) and very low incorporation of RCE, one might expect that the FTIR spectrum of CA/H/RCE would not change compared to CA/H.

### 3.3. Antibacterial Properties of the Hydrogel Film

**Figure 5:**
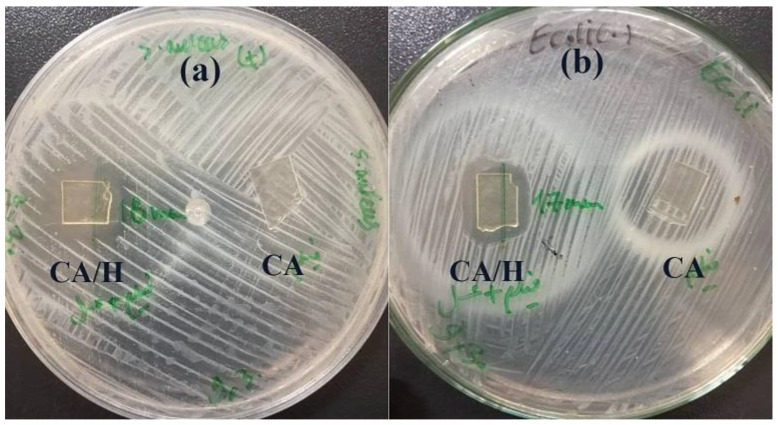
The inhibition zone of CA/H and CA hydrogel films against (**a**) *S. aureus* and (**b**) *E. coli*.

The antibacterial activity of the hydrogel films were investigated by the agar plate disk diffusion method against both Gram-positive *S. aureus* and Gram-negative *E. coli* bacteria. The results of growth inhibition of the samples are shown in Figure 5 and Table 2. Accordingly, effective growth inhibition was observed for both strains for the CA/H. The mean diameters were 18 ± 2 and 17 ± 3 mm for *S. aureus* and *E. coli*, respectively. However, the CA hydrogel film showed no zone of inhibition, and bacterial colonization was detected on this sample, suggesting that the antibacterial activity of CA/H sample is related to the incorporation of honey into the hydrogel film. It should be mentioned that incorporation of RCE resulted in no significant change in the zone of inhibition of the CA/H hydrogel film sample against either strain.

Nowadays, honey is used for the treatment of various types of infected wounds, ulcers, and burns [42,43]. El-Kased et al. prepared a honey hydrogel formulation and tested it in vivo for burn healing in mice. Their results showed that their formulation had the highest healing rate compared to commercial wound dressings. They reported that the formulation can be applied for safe and effective natural wound healing treatment [44].

### 3.4. Antioxidant Activity

**Figure 6:**
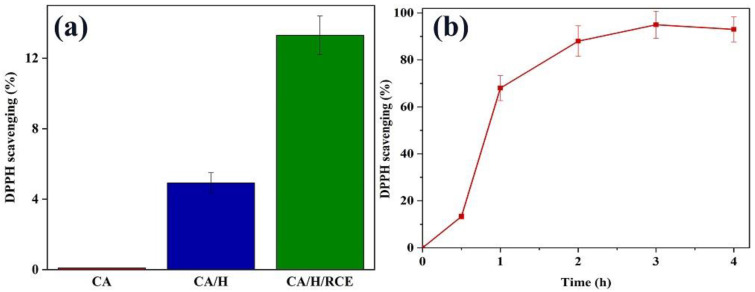
(**a**) DPPH scavenging efficiency of CA, CA/H and CA/H/RCE hydrogel films, (**b**) Time-dependent antioxidant activity of CA/H/RCE hydrogel film.

The antioxidant properties of the different samples were investigated using the DPPH assay. DPPH is known to be a stable free radical with an absorption maximum at 517 nm in its UV-Vis spectrum. DPPH molecules can be neutralized by scavenging a hydrogen atom or an electron, resulting in a color change of the solution from purple to yellow [45]. The DPPH scavenging efficiency of the samples is shown in Figure 6a. Although the CA hydrogel film showed no antioxidant activity, CA/H and CA/H/RCE exhibited DPPH scavenging efficiencies of about 4% and 13.5%, respectively. The time-dependent antioxidant activity of CA/H/RCE is shown in Figure 6b. Even though the DPPH scavenging efficiency increases with time, the slope of the curve decreases. This indicates that the reaction is complete, i.e., there is no electron or hydrogen atom left to scavenge the DPPH free radicals. 

Studies have demonstrated that the presence of reactive oxygen species (ROS) disturbs the wound healing process [46]. The reduction of the ROS level stimulates cell migration and angiogenesis, which contributes to improved wound healing [26]. In fact, ROS accumulation, more than the antioxidant capacity of a wound, is a result of a continuous inflammatory response at the wound bed, which leads to hindering phase change from inflammation to proliferation [47]. Consequently, protecting the redox balance at the wound bed by adding an antioxidant agent to the wound dressing would prevent disruption of the immune response and promote wound healing, particularly in chronic wounds [26]. Qi et al. reported a novel hydrogel dressing based on tannic acid micro particles and cationic guar gum matrix polyphenol/polysaccharide hydrogel to reduce ROS-induced oxidative damage in diabetic wounds. Both in vitro and in vivo results verified that their designed hydrogel is capable of protecting cells from ROS-induced oxidative damage [48].

### 3.5. pH-Responsive Color Change 

**Figure 7:**
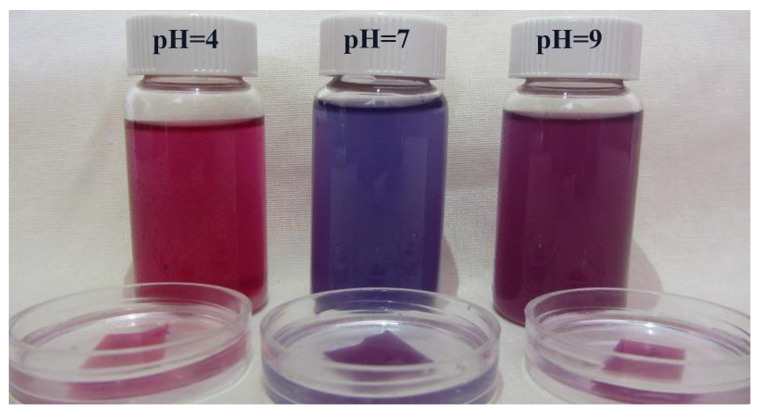
pH-responsive color change in the RCE solutions and CA/H/RCE hydrogel films in buffer solutions with different pH values.

The pH-responsiveness of the CA/H/RCE hydrogel film as well as the RCE was performed using buffers with different pH values of 4, 7, and 9. Figure 7 illustrates the resulting photograph indicating a significant color change from the acidic state to the neutral and alkaline regions for both the RCE solutions and the hydrogel films loaded with RCE. This subject demonstrates that the intensity and color shade of anthocyanins in RCE is a function of the acidity of the medium, which can be utilized as a bio-marker for developing a non-invasive wound monitoring method that prevents unnecessary dressing changes. A change from a blue to a red shade highlights a normal healing process, while a purple color change is an alarm for infection and impaired healing in chronic wounds that raise the pH to a basic level.

**Figure 8:**
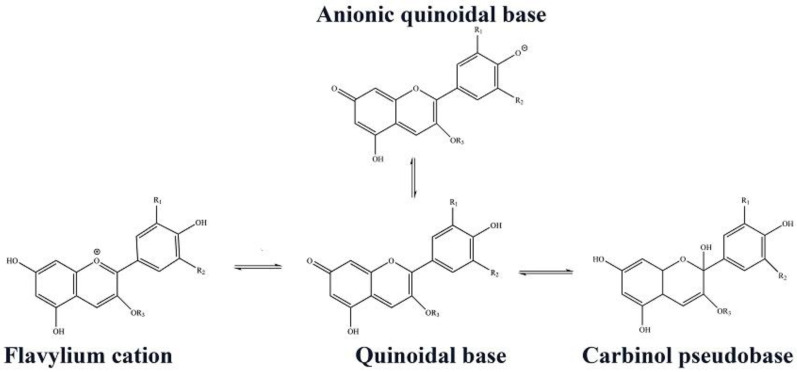
Different chemical forms of RCE anthocyanin.

The identified profile of anthocyanins from red cabbage consists of twenty different derivatives of cyanidin glucosides. This diversity is due to the number and position of methoxy and hydroxyl groups on the basic chemical skeleton [23]. The hyperchromic and bathochromic properties of RCE anthocyanins are shown in Figure 8. At low pH, the flavylium cation is predominant and causes the red color. Increasing the pH of the medium leads to a color change to a blue/purple quinoidal base. In the following, at more alkaline pH values, the colorless carbinol pseudobase will be the dominant structure [49].

### 3.6. Cell Viability

**Figure 9:**
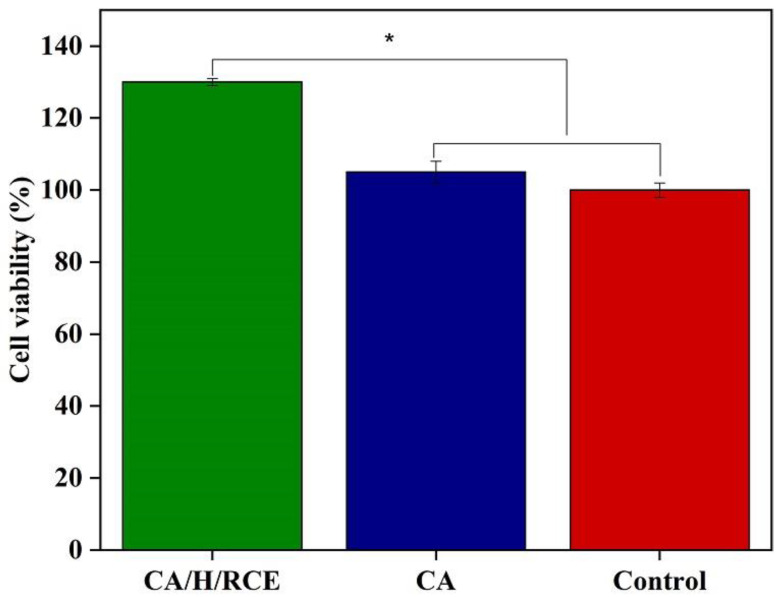
Cell viability of L929 mouse fibroblast cells exposed to CA/H/RCE and CA hydrogel films. (*, *p* < 0.05).

Cell adhesion and proliferation of CA and CA/H/RCE hydrogel films were assessed using the MTT assay (Figure 9). This method measures the reduction of the MTT tetrazolium molecule to a purple formazan component by viable cells. Thus, the level of MTT reduction, indicating normal mitochondrial activity, can be related to the level of cellular metabolism. As expected, no cytotoxic outcome was observed for the hydrogel films. Higher cell proliferation (*p* < 0.05) was observed for cells seeded on CA/H/RCE hydrogel film compared to those seeded on CA hydrogel film and the control group. No significant difference was observed between cell proliferation on CA hydrogel film and the control group.

Al-Jadi et al. investigated the stimulatory effect of honey on the proliferation of cultured fibroblasts. The authors claimed that the presence of antioxidant phenolic compounds protects cells from the toxic effects of peroxides and stimulates cell proliferation in a time- and dose-dependent manner [50]. In another study, Edgar et al. investigated the influence of natural antioxidant compounds on the proliferation and synthesis of matrix proteins by cultured normal human dermal fibroblasts. They concluded that fibroblast elastin synthesis was increased and fibroblast proliferation was enhanced in the presence of the antioxidant constituents [51]. 

## 4. Conclusions

This work aimed to produce a bio-based pH-responsive/antibacterial/antioxidant hydrogel film as a potential wound dressing. In this regard, honey and RCE were incorporated into alginate hydrogel. The mechanical properties of the hydrogel film samples showed that the incorporated honey acts as a plasticizer and enhances the mechanical properties. Moreover, a water absorption and retention study showed that the hydrogel film is able to absorb wound exudate and keep the wound moist. An antibacterial assay revealed the antibacterial activity of the honey-incorporated samples. Additionally, a DPPH assay showed the antioxidant activity of both CA/H and CA/H/RCE hydrogel films. Finally, a MTT assay confirmed that the CA/H/RCE hydrogel film promoted cell proliferation. Overall, it can be concluded that the designed CA/H/RCE hydrogel film has promising potential for wound dressings where antibacterial activity is important and antioxidant properties are required. Moreover, the pH-responsive color-change of RCE acts as a bio-marker that adds a degree of intelligence to the designed dressing. 

## Figures and Tables

**Table 1:** Mechanical properties of the honey-incorporated hydrogel (CA/H) films with different honey loading.

Sample	Honey Loading	Elastic Modulus (MPa)	Tensile Strength (MPa)	Elongation at Break (%)
CA/H	200%	5.1 ± 0.63	3.22 ± 0.91	0.69 ± 0.17
CA/H	400%	1.7 ± 0.54	6.15 ± 1.20	4.75 ± 1.26

**Table 2:** The inhibition zone of hydrogel samples against *S. aureus* and *E. coli*.

Sample	Inhibition Zone against *S. aureus* (mm)	Inhibition Zone against *E. Coli* (mm)
CA/H	18 ± 2	17 ± 3
CA/H/RCE	19 ± 3	18 ± 4

## Data Availability

Not applicable.
